# The Presence of Antineutrophil Cytoplasmic Antibodies and Antiphospholipid Antibodies in Patients with Severe Acute Respiratory Syndrome Coronavirus 2: A Case-Control Study among Sudanese Patients

**DOI:** 10.1155/2022/6511198

**Published:** 2022-12-15

**Authors:** Mohammed Shams Aldeen, Musa Logman Masaad, Ayman Azhary, Abdulomez Suliman, Mohammed Ahmed Alziber, Motasim MohammedAhmed, Farag Alla Mohamed Aman, Salahaldeen Ismail, Nadir Abuzeid, Abdualmoniem O. Musa, Samuel Tekle Mengistu, Mohammed Elfatih Hamida

**Affiliations:** ^1^Department of Medical Microbiology, Faculty of Medical Laboratory Sciences, Omdurman Islamic University, Khartoum, Sudan; ^2^Department of Medical Microbiology, Faculty of Medicine and Health Sciences, Kassala University, Kassala, Sudan; ^3^Nakfa Hospital, North Red Sea Branch of the Ministry of Health, Nakfa, Eritrea; ^4^Department of Medical Microbiology, Orotta College of Medicine and Health Sciences, Asmara, Eritrea

## Abstract

Patients infected with COVID-19 are at an increased risk for thrombosis, suggesting a possible role of COVID-19 in the induction of coagulopathy. This study aimed to investigate the presence of prothrombotic antineutrophil cytoplasmic antibodies (ANCA) and antiphospholipid antibodies (aPLs) in the course of COVID-19 infection and to correlate these markers with severity and fatality, suggesting that COVID-19-induced autoimmune thrombosis is a possible axis in the inflammatory circuit of this infection. To investigate this, we conducted a case-control study which included patients with a positive reverse transcription-polymerase chain reaction (RT-PCR) test of COVID-19 and a control group with negative COVID-19 PCR and antibody (IgG-IgM and IgA nucleoprotein) ELISA results. An indirect immunofluorescence assay using granulocyte biochips (Aesku slides from AESKU DIAGNOSTICS, Germany) was used to detect ANCA (IgG), as well as multiplex ELISA for the detection of antiphospholipid antibodies for all patients with COVID-19 and for the control group. The results revealed the detection of antiphospholipid antibodies (IgG) in one patient out of the 45 patients in the case group. 1/45(2.2%) and 7/45(15.6%) tested positive for ANCA. Five were men and two were females, with one case revealed to be positive for both aPL and ANCA. A cytoplasmic reaction on the eosinophil granulocytes was observed in 2 cases; both were positive for ANCA. Other markers (CRP, APTT, PT, INR, ESR, and neutrophil and lymphocyte counts) were included in the study, along with demographic data. No aPL or ANCA reactions were detected for any of the control groups. These findings suggest that aPL and ANCA may be induced during the course of inflammation in COVID-19 and possibly contribute to the disease's severity and mortality.

## 1. Introduction

Coronavirus disease 2019 (COVID-19) has spread throughout the world and caused a global health crisis with high mortality and morbidity since its emergence in December 2019 [1]. COVID-19 is caused by SARS-CoV-2, and although most patients recover, some develop severe and fatal complications related to pneumonia, acute respiratory distress syndrome (ARDS), thrombosis, cardiomyopathy, and acute renal injury (AKI) [2, 3]. In addition, it was observed that many patients infected with COVID-19 had developed dysregulation of the immune system and cytokine storm syndrome, which are associated with high mortality [4].

In patients with COVID-19, the outcome is believed to be determined by the regulation versus dysregulation of the inflammatory response. In the course of this inflammation, elevation of cytokines, coagulation parameters like prothrombin time (PT), partial thromboplastin activation (PTT) prolongation, increased INR [5–7], and an increase in inflammation markers such as C-reactive proteins (CRP) were observed [8]. In addition to advanced age and comorbidities, those elevated markers were also observed with mortality among COVID-19, suggesting that the fatality of COVID-19 could be in part due to thrombotic events in these individuals like deep vein thrombosis or pulmonary embolisms [9]. Including the investigation for antineutrophil cytoplasmic antibodies (ANCA) and antiphospholipid antibodies (aPLs) in patients with SARS-CoV-2 is broadening the list of possible players in the thrombosis and inflammation of COVID-19 [10, 11].

Antineutrophil cytoplasmic antibodies (ANCAs) are autoantibodies directed against various neutrophil antigens such as lactoferrin, cathepsin, elastase, myeloperoxidase (MPO), and proteinase 3 (PR3). Among them, in particular, ANCA in MPO and PR3 is associated with the development of vasculitis associated with ANCA (AAV). When neutrophils are activated by these antibodies; for example, they are believed to cause abnormal neutrophil activity and may damage the microvascular system [12, 13]. Antiphospholipid syndrome (APS) is an autoimmune disease that is associated with the formation of autoantibodies. These autoantibodies react against phospholipid-binding proteins such as beta-2-glycoprotein I (*β*2GPI) and activate endothelial cells, platelets, and neutrophils, causing thrombosis [14, 15]. The catastrophic variant of antiphospholipid syndrome is sometimes fatal and similar to the diffuse coagulopathy seen in patients with COVID-19 [16]. The majority of studies have shown that LA is known to prolong PTT and is detected in a significant proportion of COVID-19 infected patients, resulting in increased fibrinogen and factor VIII levels, elevated levels of biomarkers such as CRP, and the presence of aPLs, which can affect PTT [17]. However, aPL arise temporarily in patients with critical diseases and different viral infections due to molecular mimicry. The presence of these antibodies can cause thrombosis and makes it difficult to distinguish them from other types of thrombosis [18].

In this context, our current study tries to provide some insight into the possible association of ANCA and aPL with COVID-19 and their possible correlation with disease outcomes, suggesting a possible mechanism for autoimmune thrombosis that occurs in patients with COVID-19.

## 2. Materials and Methods

### 2.1. Study Design, Setting, and Patient Population

This is a single-center prospective case-control study between May and December 2021. A total of 90 participants were recruited from Kassala Hospital, Kassala COVID-19 isolation center, Sudan. The participants were divided into two groups: SARS-CoV-2 patients (*n* = 45) and controls (*n* = 45).

### 2.2. Definitions of Case and Control

The cohort included patients with SARS-CoV-2 (a) who came to the COVID-19 isolation center with variable infection clinical outcomes ranging from mild asymptomatic mild to server-complicated ICU patients and (b) whose COVID-19 PCR tests were positive as a result of contact tracing. The control group involved volunteers (a) who had no known history of acute, subacute, or chronic disease; (b) who did not take a particular medication; (c) who presented reasons other than infectious complaints; and (d) who gave consent to participate in the study in both the case and control groups.

The demographic characteristics and medical report forms required regarding the study for both the patient group and the healthy control group were completed by qualified physicians. Written informed consent was obtained from each participant, and the study protocol was approved by the Ministry of Health, Kassala State, Sudan. All study procedures were carried out according to the principles of the Declaration of Helsinki.

### 2.3. Laboratory Methods

From patients confirmed with positive PCR for SARS-CoV-2 in the isolation center, blood samples were collected aseptically from each participant in three different types of vacuum blood collection tubes (K2EDTA, lithium heparin, and serum) and centrifuged at 3000 rpm for 5 min at room temperature. Samples were stored at −20°C until further testing. The collection was carried out under strict COVID-19 isolation protocols [19].

### 2.4. COVID-19 Detection

The diagnosis of COVID-19 infection was confirmed in all participants by a real-time reverse transcription-polymerase chain reaction (RT-PCR) test using qTOWER³ products (Analytik Jena, Germany). A serological test for nucleocapsid protein (NP) of SARS-CoV-2 antibodies was performed using commercial enzyme-linked immunosorbent assay ELISA kits (AESKULISA®, AESKU Diagnostics) for IgG, IgA, and IgM. The assays were conducted according to the manufacturer's instructions.

### 2.5. Analytical Parameters

All participants were evaluated for biochemical indicators such as complete blood count, erythrocyte sedimentation rate (ESR), C-reactive protein (CRP), coagulation profile (PT, PTT, and INR), creatinine, urea, calcium, sodium, and potassium.

### 2.6. Detection of Antineutrophil Cytoplasmic Antibodies (ANCA)

The serum of all participants was screened for ANCA using commercial slide biochips with ethanol-fixed granulocytes from AESKU Diagnostics. The protocol was carried out on a fully automated indirect immunofluorescence (IIF) HELMED® system (AESKULISA®, AESKU Diagnostics, Wendelsheim, Germany). In brief, slides of ethanol-fixed granulocytes were incubated with diluted serum for 30 minutes at RT. After washing, the slides were treated with conjugated FITC detection antibody and incubated for 30 minutes (second incubation) and then washed with wash buffer for 5 minutes. The mounting medium was added and then covered with a coverslip; the microscopic pattern of ANCA was determined compared to positive controls for P-ANCA and CANCA according to the AESKU pattern library.

### 2.7. Detection of Antiphospholipid Antibody (aPL)

The determination of serum aPL with a 1 : 100 dilution for all participants was measured using an enzyme-linked immunosorbent assay kit (AESKULISA®, AESKU Diagnostics, Wendelsheim, Germany), which is a multiplex test for antibodies against highly purified human proteins (B2-glycoprotein 1, cardiolipin, phosphatidylcholine, ethanolamine, inositol, serine, and sphingomyelin). The protocol was performed according to the manufacturer's instructions.

### 2.8. Statistical Analysis

All data sets obtained from the study were analyzed using SPSS version 26 (SPSS Inc., Chicago, Illinois, USA) and Stata version 12.0 (Stata Corporation, College Station, TX). Continuous variables were presented as mean ± standard deviation and median (IQR), while categorical variables were provided as numbers and percentages. The Mann–Whitney *U* test, or Fisher's exact test, was used to compare the differences between the independent groups. Furthermore, the relationships between continuous variables were analyzed using Spearman correlation analysis; a chi-square test was used to analyze categorical variables. The significance level was defined as *p* < 0.05 for all analyses.

## 3. Results

### 3.1. Demographic and Clinical Characteristics of Patients

Among the 90 participants (45 cases and 45 controls), females represent 29.7% (*N* = 27) and the mean age is 43 years (SD: 16.5 years). Most of the participants are in the age range of 20 to 29 years. There are no significant differences in sex and age between cases and controls, with *p*=0.09 and *p*=0.19, respectively. Most of the participants reside in the urban areas of Kassala (85.7%) (Table 1). More than half (51.6%) have university-level education. Among chronic comorbidities, diabetes mellitus (DM) is the most common (*n* = 30, 33%). None of the controls has DM, and compared to the cases, they have a significant difference (*p* < 0.001, *χ*^2^ = 43.7). After following the cases in the hospital, death occurred in only five of the cases (*n* = 5, 5.5). Table 1 shows more information. The mean age of the dead COVID-19 cases (61 ± 12.4 years) is higher than that of the recovered COVID-19 cases (42.1 ± 16.9 years) and controls (41.9 ± 15.4 years), with *p*=0.04.

### 3.2. Hematologic Parameters, Inflammatory Markers, Electrolytes, Renal Function, and Coagulation Profile of Patients

Table 2 compares different laboratory measures between controls and the two divisions of cases, that is, recovered COVID-19 cases and dead COVID-19 cases. In terms of hematologic parameters, the groups showed a significant difference in total white blood cell (WBC) count, neutrophil count, and lymphocyte count. Leucocytosis, neutrophilia, and lymphopenia are more common among dead cases of COVID-19 than the other two. Among dead cases, the mean count of WBC is 15.2 ± 2.8 cells/*µ*L and the median is 13.9 (IQR: 12.8–18.2) cells/*µ*L, whereas in recovered COVID-19 patients, the respective measures are 12.3 ± 3.2 cells/*µ*L, 12.2 (IQR: 11.3–14.3) cells/*µ*L, and 9.8 ± 4 cells/*µ*L, 8.9 (IQR: 6.4–12.4) cells/*µ*L. The mean and median lymphocyte count among dead patients with COVID-19 is 9.2 ± 4 cells/*µ*L and 11 (5.1–12.5) cells/*µ*L, respectively. The recovered COVID-19 patients and the controls recorded relatively higher counts (*p* < 0.001). ESR and CRP are significantly higher in dead COVID-19 patients (median: 75 (IQR: 67.5–102.50) and 44 (IQR: 27.5–56.5), respectively) than in the other groups, *p* < 0.001. Although normal renal function status is recorded between controls and recovered cases, dead cases have an abnormally high record (*p* < 0.05). The median INR for dead COVID-19 patients' cases was 1.4 (IQR: 1.1–1.6) versus 0.99 (IQR: 0.9–1.1) among recovered cases and 0.95 (IQR: 0.92–1.03) in the control group, *p* = 0.025. An immunoglobulin serum assay was performed during the initial admission period on all individuals positive for RT-PCR (45 cases). IgM, IgG, and IgA were positive in 5 (10%), 13 (28), and 8 (17.4%) of the cases, respectively.

### 3.3. Autoantibodies (ANCA/aPL) Profile among Study Participants

**Figure 1:**
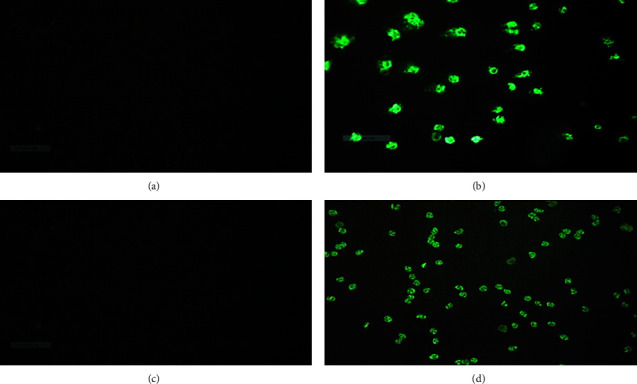
The ANCA patterns by the HELMED immunofluorescence IIF assay system via biochips of ethanol-fixed slide ethanol. (a) Negative control. (b) Positive. (c) Negative ANCA result. (d) Positive ANCA result.

**Figure 2:**
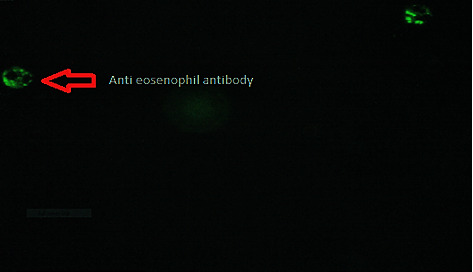
Eosinophilic cytoplasmic reaction patterns by the immunofluorescence IIF assay using granulocyte biochips from AESKU diagnostics.

Among the study participants, positivity for autoantibodies (ANCA) is observed in 7 patients. All these positive patients are cases with *p*=0.002 (*χ*^2^ = 12.27). Most of the patients are in the age group of 20–29 years (42.9%) and are male (71.4%). Among those with positive ANCA, 71.7% (*n* = 5) are diabetic (*p*=0.024), as shown in Table 3. One patient was positive for aPL besides being positive for ANCA. The detected patterns of ANCA were 4 (57.1%) cases of CANCA (Figure 1), one case of P-ANCA, and one case of atypical ANCA; a questionable reaction on eosinophils was observed in 2 patients, as shown in Figure 2. The two cases that are positive for antieosinophilic cytoplasmic antibodies had a deranged coagulation panel and elevated inflammatory markers, that is, ESR and CRP. SARS-CoV-2 nucleocapsid (NP) immunoglobulins (IgA, IgM, and IgG) were not significantly different between patients with positive autoantibodies and negative patients (Table 3).

In Table 4, the median total count of WBC among ANCA-positive individuals (15.3 (IQR: 13.9–17.8) cell/*µ*L) is higher than the median of ANCA-negative individuals (11.5 (IQR: 7.4–12.8) cell/*µ*L), with *p*=0.02. The median hemoglobin level was lower in ANCA-negative individuals (12.1 (10.5–13.3) cell/*µ*L) vs. ANCA-positive individuals (13.2 (12.3–13.9) cell/*µ*L), with *p*=0.04. Similar comparative results were recorded for mean corpuscular volume (MCV) and mean corpuscular hemoglobin (MCH), with *p*=0.03 and *p*=0.004, respectively. The ESR and CRP medians among ANCA positive individuals are 40 (IQR: 20–75) and 15 (IQR: 2–32), respectively, vs. their counterpart ANCA negative individuals recorded 25 (IQR: 20–30) and 3 (IQR: 2–8.5), with *p*=0.09 and *p*=0.11, respectively (Table 4).

### 3.4. Kaplan–Meier Analysis for Death Incidence

**Figure 3:**
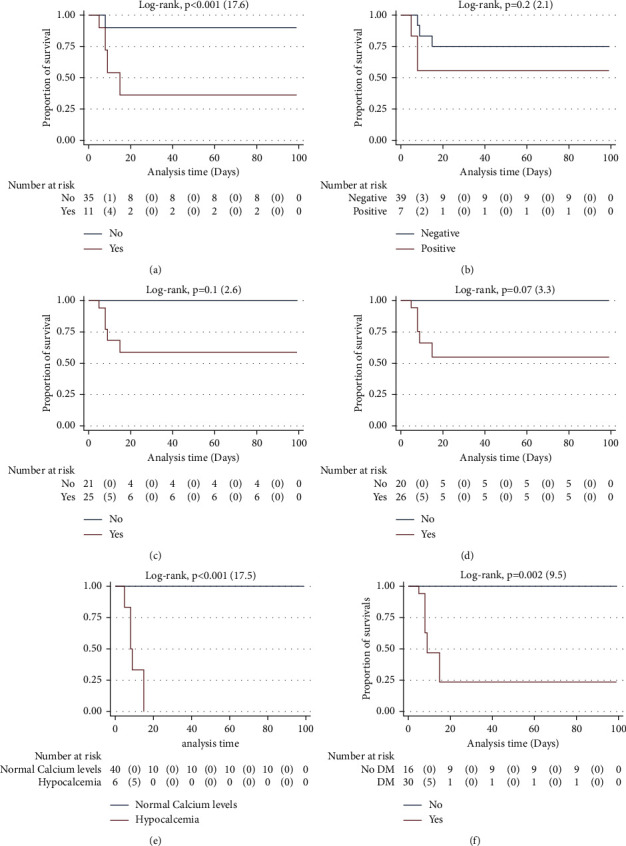
Kaplan–Meier cumulative proportion of unadjusted survival curves for SARS-CoV-2 infected individuals only (cases) between (a) those with normal and abnormal (yes) coagulation, (b) ANCA-positive and -negative patients, (c) elevated (yes) and normal levels of CRP, (d) elevated (yes) and normal levels of ESR, (e) calcium levels, and (f) presence or absence of diabetes.

Kaplan–Meier survival curves with logarithmic rank tests were constructed to compare the cumulative incidence of death from coagulation abnormalities, positivity for CRP and calcium ESR, levels among 45 patients with COVID-19, and the presence of diabetes among patients (see Figure 3). Along with the total of 1,178 days of follow-up, 5 patients (all among the COVID-19-infected individuals) died, making the incidence of death as 4.24 per 1,000 person-days. As in Figure 3(a), in general, having a coagulation abnormality was associated with shorter survival (50 (95% CI, 17–83.5 days) vs. 88.9 (95% CI, 70–107.5 days)) (log-rank test, *p* < 0.001 (17.6)). A significantly shorter mean survival duration was observed with low calcium levels, and no individual with normal calcium log-rank were dead (log rank test *p* < 0.001 (17.5)). Individuals with free DM also survived longer compared to diabetics (*p*=0.002 (9.5)).

## 4. Discussion

The incidence of autoimmune thrombosis and hemostatic changes associated with COVID-19 and other coronaviruses raises questions about possible COVID-19-related coagulopathy and thrombotic event induction [21]. ANCA and aPL have been considered as possible mechanisms that lead to proinflammatory and hypercoagulable states [22].

In our study, we screened for ANCA and aPL among a total of 90 participants, divided into 45 cases infected with COVID-19, compared to 45 healthy control individuals. Seven patients (15.6%) were found to be positive for ANCA; all of them were from the case study group (chi-square test, *p*=0.002), and one case (2.2%) was positive for aPL antibodies. This finding is close to agreement with Gelzo et al., who revealed a significant increase in ANCA levels in hospitalized patients with COVID-19 compared to asymptomatic or control individuals [23]. Sacchi et al. investigated the development of several autoantibodies in 40 patients with COVID-19 and reported positive ANCA in ten (25%) [20]. Vlachoyiannopoulos et al. tested the sera of 29 cases of severe COVID-19, revealing IFA positivity for ANCA in four cases [24]. Guven et al. tested ANCAs in 87 COVID-19 patients, revealing that eight (9.2%) had positivity in at least one ANCA test result [25]. Hamadé et al. investigated the development of aPL antibodies in 41 COVID-19 patients and reported that aPL was positive in seven (17%) [26].

The association of these autoantibodies with the clinical outcome showed that all deaths occurred only in the cases (chi-square, *p* value = 0.023); of the seven positive cases, two cases (28.6%) died while 5 (71.4%) cases recovered. Fuentes Baldarrago et al., using the APACHE II scores, demonstrated that in patients with COVID-19, there was a positive correlation between worsening of the disease state and increased NETosis, suggesting that neutrophil overactivation may play a role in disease progression [27] and may lead to the generation of ANCA seen in the serum of COVID-19 patients; a hypothesis consistent with that of O'Sullivan and Holdsworth, who suggested that dysregulation of NET formation leads to the generation of ANCA [28]; furthermore, Arcanjo et al. [29] showed for the first time that SARS-CoV-2 can activate NETosis in human neutrophils and demonstrated that this process is associated with increased levels of intracellular reactive oxygen species (ROS) [29]. Reactive oxygen species can kill pathogens directly by causing oxidative damage or indirectly, in neutrophils, by stimulating NET formation. ROS also plays a detrimental role, promoting the formation of venous thrombosis by modulating the enzymatic cascade of fibrinolysis, systems of coagulation, and the complement system. These findings undoubtedly point to the critical role of neutrophils in the pathology of infection. In other severe or persistent viral infections, neutrophil-mediated alveolar damage leads to interstitial oedema, ventilation/perfusion mismatch, and respiratory failure [29].

A recent study by Konopka et al. identified neutrophil infiltration in pulmonary capillaries in autopsy reports from patients with COVID-19. This study and others in the literature suggest an emerging possible association of ANCA and aPL with COVID-19 [30].

Our results also showed a positive case of aPL, the presence of aPL in patients with COVID-19. Following the finding of Bahramnezhad et al., which describes a case of an antiphospholipid-like condition caused by COVID-19 [31], another piece of evidence from the literature is the finding of Moon et al. [32], who presented a hypothetical model to elucidate the pathophysiology of APS in COVID-19 and described clinicopathological similarities between CAPS and severe COVID-19 [32]. In light of this evidence and our findings, COVID-19 may induce an antiphospholipid-like condition that plays a role in increasing the severity of disease manifestation; this hypothesis is strongly supported by the findings of Laura et al., who examined 106 COVID-19 patients for the presence of aPL antibodies, including 30 hospitalized cases, 47 hospitalized nonneurological COVID-19 controls, and 29 COVID-19 nonhospitalized controls, and reported a high prevalence of antiphospholipid antibodies in 72 (67.9%) of all cases and 22 (73.3%) of the 30 hospitalized neurological cases [33]. An important note in our study is to mention that the positive case of aPL is also positive for the ANCA, indicating that a wide range of autoantibodies could be induced in COVID-19 infection. Our study also demonstrated the presence of a questionable eosinophil cytoplasmic reaction, possibly being an autoantibody directed against eosinophil granules. The reaction has been observed in the serum of two patients; to our knowledge, this is the first report of the presence of possible antieosinophil cytoplasmic antibodies. There is no literature available to show the exact significance of this finding during the disease. However, in recent research, Gebremeskel et al. reported that MC-derived proteases and mediators are elevated in sera and lung tissues and suggested that MC activation and eosinophil activation are associated with COVID-19 inflammation [34]. Based on this, we can conclude that eosinophils may be involved in the pathology of the disease. However, more research is needed to determine the exact target of the cell and the role of eosinophils in this study. Kumar et al. obtained data from 33 studies, including 16003 patients, and concluded that diabetes among patients with COVID-19 is associated with a twofold increase in the severity of COVID-19 [35], compared to nondiabetics. In our study of 7 cases who tested positive for ANCA, five (71.7%) were diabetic (*p*=0.024). One of them, in addition to being positive for ANCA, was also positive for aPL antibodies and eventually died, suggesting important roles for comorbidities in COVID-19 pathogenesis.

The most common laboratory abnormalities identified in patients with COVID-19 include decreased albumin and lymphocyte count and elevated C-reactive protein (CRP), lactate dehydrogenase (LDH), erythrocyte sedimentation rate (ESR), aspartate transaminase (AST), and alanine transaminase (ALT) [36, 37]. These abnormalities are associated with worse outcomes. In our study, we compared different laboratory measures among controls and the two divisions of cases, i.e., recovered COVID-19 cases and dead COVID-19 cases. It is evident that most of the case groups had hemostatic abnormalities. The groups showed significant differences in total white blood cell count (*p*=0.002), neutrophils, and lymphocyte count (*p* < 0.001). Leukocytosis, neutrophilia, and lymphopenia are more common among dead cases of COVID-19 than the other two. ESR and CRP are much higher in dead patients with COVID-19 than in the other groups (*p* < 0.001). Although normal renal function status was recorded between controls and recovered cases, dead cases had abnormally high records (*p* < 0.05). We have found significant differences in INR between dead patients with COVID-19 and recovered cases (*p*=0.025). Supplemented by time-to-event analysis, the absence of coagulation abnormalities, normal calcium levels (in contrast to hypocalcemia), and the absence of diabetes mellitus showed survival benefits among cases. This is in line with the already known short-term prognostic indicators [35, 38, 39].

This study aimed to highlight parts of the chaotic inflammatory environment in COVID-19, possibly pointing to prognostic biomarkers and therapeutic intervention pathways, and an early attempt to put a piece on the expected long-term effects which may follow the pandemic. COVID-19 is ongoing, with almost daily emerging information. There are several limitations to this study, in addition to the small sample size. First, the study is not suitable to reveal the complete incidence of ANCA and aPL in COVID-19. The previous ANCA and aPL status of the subjects is unknown. Therefore, subclinical positivity for ANCA and aPL before the infection period cannot be ruled out, and the determination of aPL did not include IgM and IgA. Sample size may influence the statistical analysis of the ANCA positive group, which prevented further statistical investigations with analyses such as multivariate regression to better demonstrate the effects of ANCA positivity on outcomes, and in the analysis, for some patients, we only had one time point. Therefore, we recommend that more studies be conducted to investigate the relevance of the observed eosinophil cytoplasmic reaction, the target antigens, and their impact on the course of the disease. ANCA and aPL are recommended to be investigated in the study of patients with COVID-19, especially in severe cases. Autoimmune screening should be of interest clinically and research-wise in the coming years.

## 5. Conclusion

This prospective case-control study reveals that ANCA and aPL were detected in the COVID-19 infection group compared to the healthy control group. It suggests that COVID-19 induces the formation of ANCA and aPL in some patients and may contribute to their pathogenesis. Furthermore, the formation of ANCA in COVID-19 may indicate the activation and induction of the neutrophil extracellular trap in patients with COVID-19. The cytoplasmic reaction of eosinophils may suggest the presence of parallel autoantibodies to ANCAs in eosinophil granules. Further studies are needed to clarify all the different pieces of the puzzle.

## Figures and Tables

**Table 1:** Baseline sociodemographic characteristics of cases and controls.

Characteristics	Total *N* (%)	Cases *N* (%)	Controls *N* (%)	*p* value (*χ*^2^)
*Age (mean* *±* *SD)*	43 ± 16.5	44.2 ± 17.5	41.9 ± 15.5	0.194^a^
10-19	3 (3.3)	1 (2.2)	2 (4.4)	0.76 (3.4)
20–29	22 (24.2)	12 (26.1)	10 (22.)	
30–39	16 (17.6)	7 (15.2)	9 (20)	
40–49	18 (19.8)	8 (17.4)	10 (22.2)	
50–59	14 (15.4)	6 (13)	8 (17.8)	
60–60	7 (7.7)	5 (10.9)	2 (4.4)	
>70	11 (12.1)	7 (15.2)	4 (8.9)	

*Gender*				
Male	64 (70.3)	35 (78.3)	28 (62.2)	0.09 (2.8)
Female	27 (29.7)	10 (21.7)	17 (37.8)	

*Address*				
Urban	78 (85.7)	40 (89.1)	37 (82.2)	0.35 (0.89)
Rural	13 (14.3)	5 (10.9)	8 (17.8)	

*Level of education*				
No formal education	16 (17.6)	8 (17.4)	8 (17.8)	0.98 (0.15)
Primary	15 (16.5)	7 (15.2)	8 (17.8)	
Secondary	13 (14.3)	7 (15.2)	6 (13.3)	
University	47 (51.6)	23 (52.2)	23 (51.1)	

*Chronic illness*				
Diabetes mellitus	30 (33)	29 (65.2)	—	<0.001 (43.7)
Hypertension	2 (2.2)	2 (4.3)	—	0.16 (2)
Asthma	5 (5.5)	1 (2.2)	4 (8.9)	0.16 (1.9)
Seizure disorder	1 (1.1)	1 (2.2)	—	0.32 (0.98)
Renal impairment	2 (2.2)	1 (2.2)	1 (2.2)	0.99 (0)
Tuberculosis	4 (4.4)	3 (6.5)	1 (2.2)	0.32 (1)
SLE	1 (1.1)	1 (2.2)	—	0.32 (0.99)

*Final outcome*				
Recovered	86 (94.5)	40 (89.1)	45 (100)	0.023 (5.2)
Dead	5 (10.9)	5 (5.5)	—	

*Immunoglobulin's profile*				
IgM, positive	—	5 (10)	—	—
IgG, positive	—	13 (28)	—	—
IgA, positive	—	8 (17.4)	—	—

Superscript a: independent samples *t*-test. Abbreviations: IgA/G/M, immunoglobulins; SLE, systemic lupus erythematosus. Comparisons of proportions were performed using the chi-square *χ*^2^ or Fisher's exact test.

**Table 2:** Laboratory measurements of participants in cases and control participants.

Characteristics	Control (*N* = 45)		Cases (*N* = 45)				*p* value
			Dead COVID-19 patients (*N* = 5)		Recovered COVID-19 patients (*N* = 41)		
*Age (mean* *±* *SD)*	41.9 ± 15.4		61 ± 12.4		42.1 ± 16.9		0.04^a^
	Mean ± SD	Median (IQR)	Mean ± SD	Median (IQR)	Mean ± SD	Median (IQR)	

*Hematologic parameters*							
WBCs	9.8 ± 4	8.9 (6.4–12.4)	15.2 ± 2.8	13.9 (12.8–18.2)	12.3 ± 3.2	12.2 (11.3–14.3)	0.002^b^
RBCs	4.4 ± 0.5	4.4 (4–4.6)	4.1 ± 0.5	4.3 (3.7–4.5)	4.5 ± 0.6	4.7 (4.2–5)	0.08^b^
Hemoglobin (g/dL)	12 ± 1.6	11.5 (10.5–13)	11.5 ± 1.7	12.3 (9.7–12.9)	12.4 ± 1.7	12.9 (11.8–13.3)	0.22^b^
PCV	36.3 ± 4.5	35.3 (32.5–38.6)	34.5 ± 5	36.6 (29.5–38.5)	37.4 ± 5.3	38.4 (36.2–40.7)	0.17^b^
MCV (*f*L)	81.7 ± 4.4	81.8 (80.1–84.2)	83.6 ± 7.2	86.4 (76.4–89.4)	82.6 ± 5.2	83.2 (79.6–85.7)	0.6^b^
MCH	27.1 ± 2	26.8 (25.6–28.7)	27.8 ± 3	29.2 (25.2–29.9)	27.6 ± 1.7	27.4 (27–28.7)	0.26^b^
MCHC	32.9 ± 1.3	32.7 (32.3–33.5)	33 ± 1.2	32.9 (32.1–34.1)	33.3 ± 1.3	33.2 (32.3–34.3)	0.54^b^
MXD	9.4 ± 2.5	8.5 (7.5–10.7)	11.960 ± 6	10 (7.5–17.4)	8.8 ± 3.6	8 (6–12.7)	0.26^b^
RDW	13.9 ± 1.3	13.9 (13–15)	13.7 ± 1.6	13 (12.4–15.4)	13.9 ± 1.3	13.6 (13–15)	0.76^b^
Neutrophil count (K/*µ*L)	51.2 ± 14	55 (39.6–59.8)	78.6 ± 7.3	80.3 (72.5–83.9)	77.2 ± 8.3	78 (71.4–80.5)	<0.001^b^
Lymphocyte count	39.4 ± 13.3	35 (31.7–53.8)	9.2 ± 4	11 (5.1–12.5)	14 ± 6.4	13.7 (12.3–16.8)	<0.001^b^
Platelet count	307.2 ± 122.2	311 (201–381)	378.6 ± 240.9	324 (220–564.5)	296.5 ± 90.1	308 (211.5–357.5)	0.98^b^

*Inflammatory markers*							
ESR	21.9 ± 9.6	20 (20–25)	83 ± 24.6	75 (67.5–102.50)	35.5 ± 19.4	30 (20–47.5)	<0.001^b^
CRP	2.5 ± 0.6	2 (2–3)	42.4 ± 17.3	44 (27.5–56.5)	13.1 ± 15.6	3 (2–19.5)	<0.001^b^

*Electrolytes*							
Calcium	9.1 ± 0.6	9 (8.9–9.4)	7.4 ± 0.46	7.3 (6.9–7.9)	8.9 ± 0.6	9 (8.9–9.1)	0.001^b^
Sodium	137.3 ± 2.8	137 (136–140)	136.2 ± 2.3	136 (134.5–138)	137.6 ± 3.14	138 (136–139)	0.43^b^
Potassium	3.9 ± 0.3	3.9 (3.7–4.1)	4.4 ± 0.82	4.3 (3.7–5.3)	3.8 ± 0.4	3.9 (3.6–4.1)	0.24^b^

*Renal function*							
Creatinine	0.83 ± 0.3	0.8 (0.6–0.95)	4.5 ± 7.5	1.2 (1.1–9.6)	0.9 ± 0.2	0.9 (0.75–1)	0.001^b^
Urea	30.4 ± 35.7	23 (22–31)	86.8 ± 101	45 (37–157.5)	27.2 ± 8.7	25 (22–33)	0.006^b^

*Coagulation profile*							
PT	13.7 ± 1.4	13.4 (12.9–14.4)	18.98 ± 4.1	18.8 (15.4–22.7)	14.3 ± 2.3	13.9 (12.7–14.5)	0.02^b^
aPTT	35.1 ± 5	34.1 (32.1–39)	49 ± 6.7	51.1 (43–53.4)	36.7 ± 7	34.6 (32.1–38.3)	0.008^b^
INR	0.98 ± 0.1	0.95 (0.92–1.03)	1.4 ± 0.3	1.4 (1.1–1.6)	1 ± 0.2	0.99 (0.9–1.1)	0.025^b^

Superscript a: one-way ANOVA; superscript b: independent-samples Kruskal–Wallis test. Abbreviations: CRP, C-reactive protein, ESR, erythrocyte sedimentation rate, MCH, mean corpuscular haemoglobin, MCHC, mean corpuscular hemoglobin concentration, MCV, mean corpuscular volume, MXD, mixed cell count blood test, WBCs, white blood cells, RBCs, red blood cells, PCV, packed cell volume, and RDW, red cell distribution width.

**Table 3:** Autoantibodies profile of study participants.

Characteristics	Total *N* (%)	AA positive *N* (%)	AA negative *N* (%)	*p*-value (*χ*^2^)
*Case/control*				
Control	45 (49.5)	0	45 (53.6)	0.002 (12.27)
Case (recovered)	40 (45.1)	5 (71.4)	35 (42.9)	
Case (dead)	5 (5.5)	2 (28.6)	3 (3.6)	

*Age (mean* *±* *SD)*				
10–19	3 (3.3)	0	3 (3.6)	0.79 (3.13)
20–29	22 (24.2)	3 (42.9)	18 (22.6)	
30–39	16 (17.6)	1 (14.3)	15 (17.9)	
40–49	18 (19.8)	1 (14.3)	17 (20.2)	
50–59	14 (15.4)	0	14 (16.7)	
60–60	7 (7.7)	1 (14.3)	6 (7.1)	
>70	11 (12.1)	1 (14.3)	10 (11.9)	

*Gender*				
Male	64 (70.3)	5 (71.4)	58 (70.2)	0.94 (0.004)
Female	27 (29.7)	2 (28.6)	25 (29.8)	

*Address*				
Urban	78 (85.7)	6 (85.7)	72 (85.7)	1 (0)
Rural	13 (14.3)	1 (14.3)	11 (14.3)	

*Level of education*				
No formal education	16 (17.6)	3 (42.9)	13 (15.5)	0.24 (4.12)
Primary	15 (16.5)	0	15 (17.9)	
Secondary	13 (14.3)	1 (14.3)	12 (14.3)	
University	47 (51.6)	3 (42.9)	43 (52.4)	

*Symptoms*				
Fever	89 (97.8)	7 (100)	82 (97.6)	0.68 (0.17)
Cough	84 (92.3)	6 (85.7)	78 (92.9)	0.49 (0.46)
Shortness of breath	75 (82.4)	6 (85.7)	69 (82.1)	0.81 (0.06)
Sore throat	82 (90.1)	4 (57.1)	78 (92.9)	0.002 (9.2)
Headache	79 (86.8)	3 (42.9)	75 (90.5)	<0.001 (12.8)
Myalgia	63 (69.2)	4 (57.1)	59 (70.2)	0.47 (0.52)
Running nose	7 (7.7)	1 (14.3)	6 (7.1)	0.49 (0.46)

*Chronic illnesses*				
Diabetes mellitus	30 (33)	5 (71.7)	25 (29.8)	0.024 (5.1)
Hypertension	2 (2.2)	0	2 (2.4)	0.68 (0.17)
Renal impairment	2 (2.2)	0	2 (2.4)	0.68 (0.17)

*Laboratory measures*				
Hypocalcemia	8 (8.8)	3 (42.9)	5 (6)	0.001 (10.9)
Leukopenia	1 (1.1)	1 (14.3)	0	<0.001 (12.1)
Lymphopenia	48 (52.7)	7 (100)	40 (48.8)	0.009 (6.8)

*Immunoglobulin's profile*				
IgM, positive	5 (19.2)	2 (25)	3 (16.7)	0.12 (2.6)
IgG, positive	13 (50)	3 (37.5)	10 (55.6)	0.35 (0.86)
IgA, positive	8 (30.7)	3 (37.5)	5 (27.7)	0.054 (3.7)

**Table 4:** Laboratory measurements of study participants stratified by the presence or absence of autoantibodies.

	AA positive		AA negative		*p* value^a^
	Mean ± SD	Median (IQR)	Mean ± SD	Median (IQR)	
*Hematologic parameters*					
WBCs	14.2 ± 4.8	15.3 (13.9–17.8)	10.99 ± 3.8	11.5 (7.4–12.8)	0.02
RBCs	4.6 ± 0.4	4.5 (4.1–5)	4.5 ± 0.6	4.5 (4.1–4.7)	0.58
Hemoglobin (g/dL)	13.2 ± 0.8	13.2 (12.3–13.9)	12.1 ± 1.7	12.1 (10.5–13.3)	0.04
PCV	38.9 ± 2.3	38.6 (36.6–41.7)	36.5 ± 5.1	37.5 (32.5–39.5)	0.15
MCV (*f*L)	85.7 ± 4.4	86.4 (83.4–90.1)	81.9 ± 4.8	81.8 (80.1–84.5)	0.03
MCH	29.3 ± 1.5	29.5 (27.6–30.2)	27.2 ± 1.9	27.2 (26–28.5)	0.004
MCHC	34.1 ± 1.8	33.6 (32.9–36)	33 ± 1.2	33.9 (32.3–34)	0.11
MXD	10.3 ± 2.3	10.2 (8.9–12.5)	9.2 ± 3.3	8.5 (6.6–12.7)	0.38
RDW	13.1 ± 0.9	13 (12–14)	14 ± 1.3	13.6 (13–15)	0.14
Neutrophil count (K/*µ*L)	75 ± 12.6	75 (71–80.3)	63.7 ± 17.6	68.9 (48.2–79.8)	0.17
Lymphocyte count	17.1 ± 12.1	13.7 (11.3–17.5)	27.1 ± 16.7	21.2 (13.6–41.4)	0.13
Platelet count	286.9 ± 49.5	292 (233–330)	307.9 ± 121.5	311 (208.5–381)	0.64

*Inflammatory markers*					
ESR	44.3 ± 25.2	40 (20–75)	30.3 ± 20.4	25 (20–30)	0.09
CRP	18.6 ± 17.3	15 (2–32)	8.7 ± 14.2	3 (2–8.5)	0.11

*Electrolytes*					
Calcium	8.1 ± 1.1	9 (6.9–9)	9 ± 0.6	9 (8.9–9.1)	0.16
Sodium	135.4 ± 2.7	136 (132–138)	137.6 ± 2.9	137 (136–140)	0.06
Potassium	3.8 ± 0.4	3.8 (3.6–4)	3.9 ± 0.4	3.9 (3.7–4.1)	0.56

*Renal function*					
Creatinine	1 ± 0.2	1 (0.9–1.2)	1.1 ± 1.8	0.9 (0.7–1)	0.03
Urea	29.7 ± 13.5	29 (17–45)	32.3 ± 37.2	24 (22–33)	0.64

*Coagulation profile*					
PT	14.5 ± 2.2	14.4 (12.4–17.3)	14.2 ± 2.3	13.4 (12.9–14.5)	0.86
PTT	38.7 ± 7.4	35.8 (34.2–46.5)	36.4 ± 6.7	34.4 (32.1–39)	0.34
INR	1 ± 0.17	1.03 (0.9–1.3)	1.02 ± 0.2	0.95 (0.92–1.1)	0.86

Superscript: a; independent sample Mann–Whitney *U* test. Abbreviations, AA: autoantibodies, CRP, C-reactive protein, ESR, erythrocyte sedimentation rate, MCH, mean corpuscular haemoglobin, MCHC, mean corpuscular hemoglobin concentration, MCV, mean corpuscular volume, MXD, mixed cell count blood test, WBCs, white blood cells, RBCs, red blood cells, PCV, packed cell volume, and RDW, red cell distribution width.

## Data Availability

All data sets used for this work are accessible from the corresponding author on reasonable request.

## Abbreviations

AA: Autoantibodies
AAV: ANCA-associated vasculitis
AKI: Acute renal injury
ANA: Antinuclear antibodies
ANCA: Antineutrophil cytoplasmic antibody
APL: Antiphospholipid antibodies
APS: Antiphospholipid syndrome
CANCA: Cytoplasmic ANCA
COVID-19: Coronavirus disease 2019
CRP: C-reactive protein
DM: Diabetes mellitus
ELISA: Enzyme-linked immunosorbent assay
ESR: Erythrocyte sedimentation rate
ICU: Intensive Care Unit
IgA: Immunoglobulin A
IgG: Immunoglobulin G
IgM: Immunoglobulin M
INR: International normalized ratio
MCH: Mean corpuscular haemoglobin
MCHC: Mean corpuscular hemoglobin concentration
MCV: Mean corpuscular volume
MPO: Myeloperoxidase
pANCA: Perinuclear ANCA
PR3: Proteinase 3
IIF: Indirect immunofluorescence
PT: Prothrombin prolongation
APTT: Activated partial thromboplastin time
RT-PCR: Reverse transcription-polymerase chain reaction
SARS-CoV-2: Severe acute respiratory syndrome coronavirus 2.
